# One-step reconstruction of IVC and right hepatic vein using reversed auto IVC and left renal vein graft

**DOI:** 10.1016/j.ijscr.2019.03.004

**Published:** 2019-03-16

**Authors:** Susumu Eguchi, Shinichiro Ono, Akihiko Soyama, Saeko Fukui-Araki, Yuriko Isagawa-Takayama, Masaaki Hidaka, Tomohiko Adachi, Takashi Hamada, Yu Huang, Kengo Kanetaka, Mitsuhisa Takatsuki

**Affiliations:** Department of Surgery, Nagasaki University Graduate School of Biomedical Sciences, Nagasaki, Japan

**Keywords:** Extracorporeal resection, Inferior vena cava, Left renal vein, Hepatic vein

## Abstract

**Introduction:**

A malignant tumor invading the inferior vena cava (IVC) and concomitant with 3 hepatic venous involvement still remains surgical therapeutic challenge.

**Presentation of case:**

A 79-year-old male presented with liver dysfunction and was eventually diagnosed with an intrahepatic huge IHCC originating at the level of confluence of 3 hepatic veins to the IVC, extending to the right hepatic vein (HV). Under extracorporeal circulation, the liver with the IVC, portal vein, hepatic artery and common bile duct were removed *en bloc*. In the back table, the left liver lobe was resected including the tumor and IVC, and the right HV was reconstructed using the patient’s reversed left renal vein (LRV) and IVC graft. Subsequently, the patient’s right liver lobe with the reversed LRV/IVC graft was transplanted back into the patient using a partial liver transplant technique. Liver enzymes gradually came down to the value within normal range on postoperative day 13 with the patency of the right HV. However, the patient expired on POD 16 because of sudden septic complications.

**Discussion:**

This technique could be one of the options to reconstruct the HV/IVC when a tumor invades all 3 HVs.

**Conclusion:**

To the best of our knowledge, this is the first report of such HV reconstruction in combination with extracorporeal resection of huge liver cancer in updated world literature.

## Highlights

• The left liver lobe was resected including the tumor and IVC ex vivo, and the right hepatic vein were reconstructed using the reversed patient’s left renal vein (LRV) and IVC graft.
• The patient’s right liver lobe with the reversed LRV/IVC graft was transplanted back into the patient using a partial liver transplant technique.

## 1 Introduction

The malignant tumor invading the inferior vena cava (IVC) concomitant with hepatic venous involvement still remains a surgical therapeutic challenge. Especially for metastatic colorectal cancer or intrahepatic cholangiocellular carcinoma (IHCCC), the treatment is generally complete resection, which can provide long-term survival [1]. There have been several reports that showed a successful removal of an IVC and hepatic vein using artificial vessels [[2], [3], [4]]. However, there are a few reports available regarding successful removal of a far advanced IVC and hepatic vein reconstruction with one-step reconstruction using patient’s IVC together with the left renal vein. We herein describe the first case of IHCCC that had invaded the IVC and the right hepatic vein (RHV), which was successfully resected *ex vivo* and reconstructed with auto venous graft combining the ICV and the left renal vein (LRV). Specifically, the procedure consisted of extracorporeal resection, back table reconstruction of the right hepatic vein and IVC with auto IVC/LRV graft and liver autotransplantation.

The work has been reported in line with the SCARE criteria [5].

## 2 Presentation of case

**Fig. 1:**
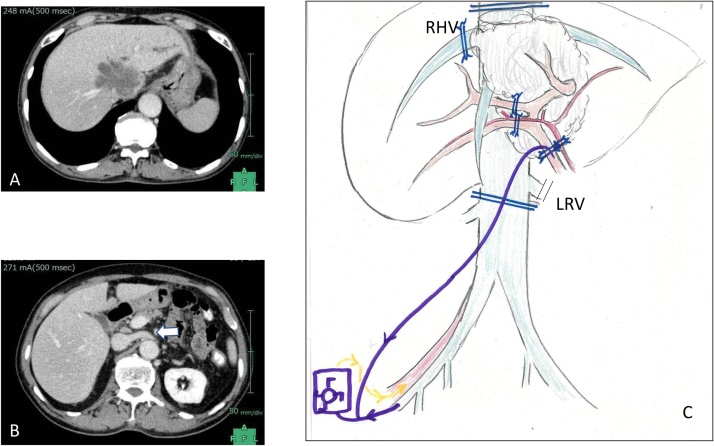
**A**: Computed tomography showing ICC located on the 3 major hepatic veins. **B**: The left renal vein was intact. **C**: Schematic drawing of whole related anatomy before the surgery.

**Fig. 2:**
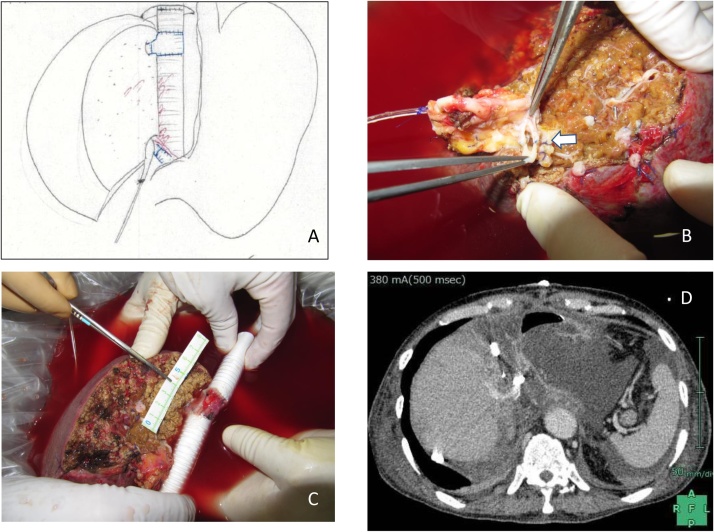
**A**: Reconstructed right hepatic vein with the left renal vein/ IVC graft. **B**: The anterior and posterior branches of the portal veins were combined. (arrow) **C**: Schematic drawing of the entire reconstruction after the surgery. **D:** The reconstructed hepatic vein with LRV as well as IVC were patent.

A 79-year-old male presented with liver dysfunction and was eventually diagnosed with an intrahepatic huge IHCC originating at the level of the confluence of the 3 hepatic veins to the IVC, extending to the right hepatic vein (Fig. 1A–C). Since his liver function was within normal range using the Child-Pugh A classification and there was no distant metastasis, he was scheduled for extensive hepatic resection ex vivo and autograft reconstruction of the resected RHV and IVC with inverted IVC/LRV graft and transplant back (Fig. 2A).

In a basin with ice-cold histidine-tryptophan-ketoglutarate (HTK) solution, the left hepatic lobe and the IVC including the tumor were resected with the right hepatic vein with a CUSA. In the back table procedure, the stump of the right hepatic vein was anastomosed to the left hepatic vein with IVC after placing it in the reversed position, which was 10 mm in diameter in an end-to-end manner (Fig. 2B) to ensure the outflow of the liver. The two portal veins were combined into one (Fig. 2C). The anastomosis was made with 4-0 non-absorbable Polypropylene (Prolene^®^) running sutures. Also, a PTFE graft was placed supra and infra the hepatic IVC to adjust the gap for future anastomosis to the graft IVC.

Right liver lobe autotransplantation that was anastomosed with graft IVC/LRV was performed using the same procedure as for conventional whole-liver transplantation. The duration of the entire operation was 1,114 min with anhepatic time of 289 min. Since the duration under extracorporeal circulation was 507 min, the total blood loss was 20,343 cc which was enhanced by heparin administration for extracorporeal circulation but recovered using an autologous blood recovery system. The postoperative course was uneventful in the intensive care unit, without any significant derangement in the liver dysfunction.

The pathological diagnosis indicated IHCCC. The patency of the hepatic vein was ensured, as confirmed by contrast-enhanced CT (Fig. 2D) on postoperative day 3. Liver enzymes had gradually come down to the value within normal range on postoperative day 13. However, the patient expired on postoperative day 16 after the surgery because of a sudden septic complication.

## 3 Discussion

We herein report a case of huge centrally located IHCC involving three hepatic veins and IVC, which was successfully removed extracorporeally followed by reconstruction with the reversed LRV/IVC graft. There have been several reports of very aggressive hepatectomy using a back table procedure for advanced liver tumors, including those using liver transplantation techniques [6,7]. When the tumor extends to the level of the orifice of the hepatic veins, a tumor removal with right or left hepatectomy can be performed. To reconstruct the right hepatic vein and IVC simultaneously, we used orifice of the left renal vein which was still connected with the IVC and inverted its direction.

Liver autotransplantation has been reported after extracorporeal hepatectomy including our own reports. However, there have been no reports of the resection of a liver tumor involving the 3 hepatic veins and IVC simultaneously [8]. Another option to resect the RHV/IVC-involving tumor could be an in situ excision with cold preservation through the portal vein with liver in place, called hypothermic preservation technique [9,10]. However, it has been thought that it would be difficult to reconstruct all three hepatic veins with the prosthetic IVC in situ.

To the best of our knowledge, this is the first report of such reconstruction in combination with extracorporeal resection of huge liver cancer in the updated world literature. Only the liver transplant technique allowed us to complete the total excision of the tumor to potentially provide long-term survival.

## 4 Conclusion

We experienced a rare case of advanced centrally located IHCC involving the RHV and the IVC, for which extracorporeal resection was followed by liver autotransplantation with auto LRV/IVC graft substituted for the original RHV/IVC. This technique could be one of the options to reconstruct RHV/IVC when a tumor invades all three hepatic veins.

## Conflicts of interest

No conflict of interest.

## Source of funding

No funding on this study.

## Ethical approval

Approval to publish this case report was waived by the institution.

## Consent

Written informed consent for publication of this case report and accompanying images was obtained from the patient. A copy of the written consent is available for review by the Editor-in-Chief of this journal, on request.

## Author contribution

SE, SO, AS, MH, SAF, YIT and MT performed the surgery and were responsible for the care of the patient. SE, KK, TH and YH designed and drafted the manuscript. MT and TA reviewed and revised the manuscript.

## Registration of research studies

This is not systematic review or meta-analysis. Also this is not randomised clinical trial.

## Guarantor

The Guarantor is Mitsuhisa Takatsuki MD, PhD.

## Submission declaration

The authors declare that the work described has not been published previously, that it is not under consideration for publication elsewhere, that its publication has been approved by all authors and either tacitly or explicitly by the responsible authorities where the work was carried out, and that, if accepted, it will not be published elsewhere—including electronically in the same form in English or any other language—without the written consent of the copyright holder.

## Provenance and peer review

Not commissioned externally peer reviewed.
